# An evaluation of exclusionary medical/psychiatric conditions in the definition of chronic fatigue syndrome

**DOI:** 10.1186/1741-7015-7-57

**Published:** 2009-10-12

**Authors:** James F Jones, Jin-Mann S Lin, Elizabeth M Maloney, Roumiana S Boneva, Urs M Nater, Elizabeth R Unger, William C Reeves

**Affiliations:** 1 Chronic Viral Diseases Branch, Coordinating Center for Infectious Diseases, Centers for Disease Control and Prevention, 1600 Clifton Road, MS A15, Atlanta, GA 30333, USA; 2 University of Zurich, Department of Psychology, Clinical Psychology and Psychotherapy, Binzmuehlestrasse 14/Box 26, Zurich 8050, Switzerland

## Abstract

**Background:**

The diagnosis of chronic fatigue syndrome (CFS) in research studies requires the exclusion of subjects with medical and psychiatric conditions that could confound the analysis and interpretation of results. This study compares illness parameters between individuals with CFS who have and those who do not have exclusionary conditions.

**Methods:**

We used a population-based telephone survey of randomly selected individuals, followed by a clinical evaluation in the study metropolitan, urban, and rural counties of Georgia, USA. The medical and psychiatric histories of the subjects were examined and they underwent physical and psychiatric examinations and laboratory screening. We also employed the multidimensional fatigue inventory (MFI), the medical outcomes survey short form-36 (SF-36) and the US Centres for Disease Control and Prevention symptom inventory (SI).

**Results:**

Twenty-nine percent (1,609) of the 5623 subjects who completed the detailed telephone interview reported exclusionary diagnoses and we diagnosed an exclusionary condition in 36% of 781 clinically evaluated subjects. Both medical and psychiatric exclusionary conditions were more common in women, blacks and participants from rural areas. Subjects with and without exclusions had similar levels of fatigue and impairment as measured by the MFI and SF-36; those with CFS-like illness (not meeting the formal CFS definition) were more likely to have an exclusionary diagnosis. After adjusting for demographics, body mass index, fatigue subscales, SF-36 subscales and CFS symptoms, CFS-like illness did not remain significantly associated with having an exclusionary diagnosis.

**Conclusion:**

Medical and psychiatric illnesses associated with fatigue are common among the unwell. Those who fulfill CFS-like criteria need to be evaluated for potentially treatable conditions. Those with exclusionary conditions are equally impaired as those without exclusions.

## Background

The diagnosis of chronic fatigue syndrome (CFS) for research and clinical purposes is based on a clinical definition: persistent or relapsing fatigue of at least 6-months' duration, that is not alleviated by rest and that causes a substantial reduction in activities; a fatigue that cannot be explained by medical or psychiatric conditions and must be accompanied by at least four of the eight case defining symptoms (unusual post exertional malaise, impaired memory or concentration, unrefreshing sleep, headaches, muscle pain, joint pain, sore throat and tender cervical nodes) [1].

Since CFS definitions were originally formulated for research purposes, the exclusion of active medical and psychiatric illnesses or diseases associated with fatigue was considered necessary for two reasons [1-5]. First, some of those suffering such symptoms have an explanation for their fatigue; and, secondly, mislabelling a condition that would respond to specific therapy (i.e., anaemia or hypothyroidism) as CFS would not be appropriate. In addition, those who had psychotic psychiatric illnesses might not be able to realistically report their symptoms and levels of function. Thus, certain diseases such as hypothyroidism and diabetes mellitus are considered exclusionary until adequately treated [4]. Other diseases with core symptoms that are also seen in CFS - for example, a major depressive disorder with melancholic features (MDDM) or chronic hepatitis C - were considered permanent exclusions because of their persistent or recurrent course.

In addition to its use in research, the definition is widely used for clinical purposes. The clinical application has been hampered because it gives practitioners little guidance on how to determine when a patient is sufficiently impaired or symptomatic to warrant the diagnosis and when to include patients with adequately treated 'exclusionary' diseases who may continue to have symptoms and consequences compatible with CFS. The first issue was partly addressed by the application of empiric methods to the diagnostic process [6]. However, the problem of how to handle exclusionary diseases remained unanswered. For example, patients with hypothyroidism or systemic lupus erythematosus that is 'under control' or inactive, based on physical examinations and laboratory evaluations, may still fulfill the criteria for CFS. Clinically, they are frequently managed as CFS patients, raising the question of whether those who would meet the research criteria for CFS, except for the single fact that they have an exclusionary condition, are similar to those who fully meet the CFS case definition criteria of fatigue severity, impairment and specific symptoms and, by inference, of the underlying pathophysiology of fatigue. It is also recognized that, while the illnesses are considered exclusionary because of their frequent association with fatigue, many with those diagnosed do not have fatigue.

The purpose of this analysis is to describe the prevalence of exclusionary diagnoses identified in a population-based surveillance study of fatigue illnesses and to compare those with and without such conditions in terms of: (1) demographics; (2) specific exclusionary categories; (3) the presence of CFS case-definition criteria; and (4) levels and characteristics of fatigue and impairment. The frequency with which potentially treatable conditions were identified in subjects otherwise meeting research criteria for CFS emphasizes the need for clinicians treating patients presenting with unexplained fatigue to consider these illnesses in the differential diagnosis.

## Methods

This study was approved by the US Centers for Disease Control and Prevention (CDC) Institutional Review Board, as required by Department of Health and Human Services regulations. All participants were volunteers who gave their informed consent. The study was conducted in English. Non-English speaking respondents were excluded.

### Study design

The study design was described in detail by Reeves *et al*. [7]. Subject participation required contact with a screening interviewer, followed by a detailed telephone interview and a clinic visit. Between September 2004 and July 2005 Georgia residents between the ages of 18 and 59 were identified for participation in a telephone screening survey conducted in the metropolitan, urban and rural areas of Georgia [8,9]. A household informant (≥ 18 years) identified as *unwell *any household members who had had at least one of the four most common CFS defining symptoms (fatigue, cognitive impairment, un-refreshing sleep, muscular or joint pain) for ≥ 1 month and as *well *those who had experienced none of these problems for ≥ 1 month.

All those identified as unwell with fatigue, as well as randomly selected persons identified as well and as unwell without fatigue, were asked to complete a detailed computer assisted telephone interview (CATI). On the basis of the telephone interview respondents were classified as: (1) having a medical or psychiatric condition considered exclusionary; (2) having a CFS-like illness characterized by severe fatigue lasting for 6 months or longer that was not alleviated by rest, that caused a substantial reduction in occupational, educational, social or personal activities and that was accompanied by at least four of the CFS case defining symptoms (tentatively CFS pending clinical evaluation); (3) having prolonged (≥ 1 month) or chronic (≥ 6 months) unwellness with or without fatigue, but not meeting criteria for CFS; or (4) being well. All respondents classified as CFS-like (*n *= 469), those classified as chronically unwell (*n *= 505), and the randomly selected well (*n *= 641) matched to the CFS-like classification on age, sex and geographic area were invited to a 1-day clinic where they were given a physical examination, a psychiatric interview, laboratory screening and completed self-administered questionnaires. Final classification categories include: (1) CFS; (2) insufficient fatigue (ISF; subjects who have been ill for > 6 months, but do not fulfill all criteria for CFS); (3) non-fatigued (NF); and (4) subjects who appeared in each category but for an exclusionary diagnosis.

### Data collection

Demographic data (age, sex, race, geographic strata) and past medical and psychiatric history data were obtained via telephone interviews and confirmed at clinic by self-administered questionnaires. Height and weight were measured at the clinic; body mass index (BMI) was calculated as the ratio of weight in kilograms to height in metres squared. To complete the screening for existing, but unidentified, medical and psychiatric conditions, participants reviewed current medications and medical history with clinic personnel and had a complete physical examination with laboratory tests recommended for the evaluation of possible CFS. In addition, a psychiatric interview was conducted using the structured clinical interview for disorders described in the forth edition of the *Diagnostic and Statistical Manual for Mental Disorders *(SCID DSM-IV) [10]. A review committee of CDC and Emory University physicians and psychologists reviewed all the data collected at the clinic visits to determine the presence of medical and psychiatric conditions considered exclusionary for CFS.

We used the multidimensional fatigue inventory (MFI) [11] to assess the fatigue status. Functional impairment was assessed by the medical outcomes survey short form-36 (SF-36) [12]. The SF-36 also yields two summary scores that reflect the two-dimensional factor structure underlying the eight subscales: a physical component summary (PCS) score and a mental component summary (MCS) score [13]. Nichol's health utility index (HU12) was generated from eight subscales of SF-36 [14]. We used the CDC symptom inventory (SI) [15] to evaluate occurrence, frequency and severity of the eight CFS-defining and 10 accompanying symptoms.

### Statistical analyses

General linear models were performed in order to examine the bivariate associations between health outcome measures (fatigue, functional impairments and symptoms) and the following covariates: (1) diagnostic subgroups (CFS-like, unwell or well); (2) exclusion status (included or excluded); (3) demographics such as sex, age, race or geographic stratum; (4) BMI. Tukey-Kramer tests were employed to test significance of *ad hoc *comparisons. We used logistic regression to generate odds ratios (OR) as measures of the association between the inclusion/exclusion status and the demographic factors and qualitative health status. A multivariate logistic regression model was used to examine the likelihood of a subject being excluded with the adjustment of all other covariates in the model. All tests of significance were two-sided with the alpha level set at 0.05. The analyses were performed using SAS version 9.1 (SAS Institute, Inc., Cary, NC, USA).

## Results

### Demographic characteristics

The study sample of 5,623 subjects at a CATI evaluation (the detailed interview) were primarily female (64%), white (65%), living in rural or urban areas (80%) with a mean age of 41.6 years (data statistics not shown). Similar distributions were present in the 781 subjects seen in the clinic whose mean age was 43.5 years (see Table 1, Part A) - the majority were female (76%), white (71%) and living in rural or urban areas (83%).

**Table 1:** Characteristics of clinically evaluated subjects

**Characteristics**			**Exclusionary Condition**				Odds ratio (OR)*	
	**All**		**Yes**		**No**		**Unadjusted Ors**	**Adjusted Ors**
	***n *= 781**		***n *= 280 (36%)**		***n *= 501 (64%)**		**(95% CI)**^**†**^	**(95% CI)**
**Part A** Classification								
CFS-like	291	(37.26)	141	(48.45)	150	(51.55)	**3.25 (2.03-4.80)**	1.50 (0.79-2.87)
Unwell	267	(34.19)	89	(34.19)	178	(66.67)	**1.73 (1.15-2.59)**	0.89 (0.53-1.52)
Well	223	(28.55)	50	(22.42)	173	(77.58)	1.00	1.00
Age (years), mean (SD)	43.50	(10.36)	43.10	(10.39)	43.73	(10.35)	0.94 (0.81-1.09)	0.83 (0.70-1.00)
Sex								
Male	186	(23.82)	71	(38.17)	115	(61.83)	1.14 (0.81-1.60)	1.44 (0.96-2.16)
Female	595	(76.18)	209	(35.13)	386	(64.87)	1.00	1.00
Race								
Black	195	(24.97)	91	(46.67)	104	(53.33)	**1.86 (1.34-2.60)**	**1.89 (1.24-2.88)**
Other	35	(4.48)	13	(37.14)	22	(62.86)	1.26 (0.62-2.56)	1.12 (0.51-2.45)
White	551	(70.55)	176	(31.94)	375	(68.06)	1.00	1.00
Residential areas								
Rural	383	(49.04)	144	(37.60)	239	(62.40)	**1.81 (1.16-2.82)**	**2.02 (1.20-3.39)**
Urban	266	(34.06)	103	(38.72)	163	(61.18)	**1.90 (1.19-3.02)**	**1.96 (1.16-3.29)**
Metropolitan	132	(16.90)	33	(25.00)	99	(75.00)	1.00	1.00
BMI (kg/m^2^), mean (SD)	28.08	(5.34)	28.76	(5.52)	27.70	(5.20)	**1.22 (1.05-1.41)**	1.02 (0.86-1.22)

**Part B** MFI							Per SD increment	Per SD increment
General fatigue	12.90	(4.69)	14.10	(4.36)	12.22	(4.74)	**1.52 (1.30-1.78)**	1.23 (0.87-1.74)
Physical fatigue	10.86	(4.37)	12.05	(4.32)	10.20	(4.25)	**1.54 (1.32-1.79)**	1.06 (0.78-1.44)
Reduced activity	9.24	(4.16)	10.13	(4.33)	8.75	(3.98)	**1.39 (1.20-1.61)**	0.93 (0.72-1.19)
Reduced motivation	9.58	(3.90)	10.60	(4.12)	9.01	(3.66)	**1.51 (1.30-1.76)**	1.21 (0.92-1.59)
Mental fatigue	10.94	(4.54)	12.09	(4.50)	10.29	(4.43)	**1.50 (1.29-1.74)**	1.12 (0.89-1.40)
SF-36							Per SD increment	Per SD increment
Physical Functioning	75.64	(24.03)	67.57	(26.41)	80.11	(21.35)	**1.68 (1.44-1.95)**	0.62 (0.34-1.10)
Role physical	41.41	(41.60)	27.14	(37.45)	49.50	(41.68)	**1.78 (1.52-2.09)**	0.70 (0.50-0.99)
Bodily pain	60.61	(26.63)	52.35	(26.93)	65.23	(25.34)	**1.65 (1.41-1.92)**	0.66 (0.41-1.07)
Social functioning	71.32	(27.78)	62.28	(29.17)	76.37	(25.65)	**1.67 (1.43-1.94)**	1.08 (0.70-1.68)
Mental health	68.50	(22.23)	62.29	(23.05)	71.98	(20.99)	**1.55 (1.33-1.80)**	1.11 (0.63-1.94)
Role emotional	49.12	(43.31)	37.74	(41.49)	55.49	(43.05)	**1.52 (1.31-1.77)**	1.08 (0.78-1.50)
Vitality	47.77	(28.01)	41.39	(27.03)	51.38	(27.94)	**1.44 (1.24-1.67)**	**1.61 (1.02-2.53)**
General health	61.87	(23.57)	55.24	(23.88)	65.58	(22.58)	**1.56 (1.34-1.82)**	0.98 (0.64-1.52)
PCS	46.28	(10.72)	43.07	(11.64)	48.08	(9.72)	**1.60 (1.38-1.86)**	1.50 (0.40-5.68)
MCS	44.67	(12.79)	41.27	(13.63)	46.58	(11.89)	**1.52 (1.31-1.76)**	0.68 (0.22-2.07)
HUI2†	76.14	(14.61)	70.75	(14.80)	79.16	(13.61)	**1.82 (1.56-2.13)**	Not included^§^
SI							Per SD increment	Per SD increment
SI_CFS	23.13	(20.58)	28.13	(21.45)	20.34	(19.56)	**1.45 (1.25-1.68)**	0.78 (0.56-1.08)
SI_NCFS	19.89	(18.09)	24.44	(20.21)	17.34	(16.26)	**1.48 (1.27-1.71)**	1.04 (0.79-1.37)

*Adjusted ORs were obtained for the multiple logistic regression models including all variables in the entire table; ORs with *P*-values < 0.05 are in bold font.

^† ^Nichole's Health Utility Index 2 (HUI2) was included in the multivariate model because it is a linear combination of eight of the Medical Outcomes Survey Short Form-36 (SF-36) subscales and strongly correlated with physical component summary (PCS) and mental component summary (MCS).

CFS = chronic fatigue syndrome; CI = confidence interval; SD = standard deviation; BMI = body mass index; MFI = multidimensional fatigue inventory; SI_CFS = symptom inventory summary score of 8 CFS symptoms; SI_NCFS = symptom inventory summary score of non-CFS symptoms

### Exclusionary conditions

Based on the responses of those completing the detailed telephone interview: 907 met criteria of the 1994 CFS case definition [1] and, prior to a clinical examination, we classified them as CFS-like; 3203 reported prolonged (≥ 1 month) or chronic (≥ 6 months) unwellness, with or without fatigue, but not meeting criteria for CFS; and 1513 were well. In all, 1609 (28.6%) of those interviewed reported a medical or psychiatric condition considered exclusionary for CFS (Table 2). Four hundred and forty-one (48%) of those classified as CFS-like reported such conditions, as did 982 (37%) of the unwell and 186 of the well (9%). Most (1341; 83%) of the excluded individuals identified by the detailed interview had exclusionary medical reasons for their exclusion (Table 3). About 60% (814) of those excluded at this stage were reported to be: morbidly obese (BMI ≥ 40); suffering narcolepsy or sleep apnea; had been pregnant within the preceding year; had HIV/AIDS; or had undergone an organ transplantation. These exclusions rendered the individual ineligible for clinical evaluation and they were, therefore, not evaluated further.

**Table 2:** Excluded participants by telephone interview classifications

**Telephone interview**												
	**All**	Excluded (*n *= 1609)*										
	
			**Medical (only)**		**Psychiatric (only)**		**Medical and psychiatric**		**Missing data**		**Subtotal (%)**	
			
			***n***	**(%)**	***n***	**(%)**	***n***	**(%)**			***n***	**(%)**
			
CFS-like	910	48%	350	79.4	60	13.6	31	7.0	0	0	441	27.4
Unwell	2635	37%	827	84.2	105	10.7	50	5.1	0	0	982	61.0
Well	2085	9%	164	88.2	16	8.6	6	3.2	0	0	186	11.6
Total	5630	29%	1341	83.3	181	11.3	87	5.4	0	0	1609	100

Clinic evaluation												

CFS-like	291	49%	57	40.1	62	43.7	22	15.5	1	0.7	142	50.4
Unwell	267	43%	56	49.6	46	40.7	11	9.7	1	0.9	114	40.5
Well	223	12%	20	76.9	6	23.1	0	0.0	0	0.0	26	9.2
Total	783	36%	133	47.2	114	40.4	33	11.7	2	0.7	282	100

*Row percentages are listed unless other indicated

CFS = chronic fatigue symptoms

**Table 3:** Numbers and types of exclusionary conditions

	**Telephone interview** (*n *= 5558*)		**Clinic evaluation** (*n *= 781*)	
	***n***	**(%)**	***n***	**(%)**
**Medical**				
Morbid obesity (BMI> = 40 kg/m^2^)	335	6.0	0	0
Pregnancy within the past year	282	5.1	0	0
HIV or AIDS	33	0.6	0	0
Organ transplantation	14	0.3	0	0
Sleep disorders	289	5.2	1	0.1
Neurological	^†^105	1.9	4	0.5
Autoimmune/inflammatory	^‡^88	1.6	33	4.2
Cardiovascular	77	1.4	12	1.5
Infection	^§^46	0.8	1	0.1
Cancer	28	0.5	0	0
Diabetes mellitus Type 1	22	0.4	22	2.8
Blood disorders	23	0.4	32	4.1
Lung disease	19	0.3	6	0.8
Liver	16	0.3	1	0.1
Renal	11	0.2	3	0.4
Surgery	10	0.2	0	0
Thyroid/pituitary	5	0.1	42	5.4
Other	72	1.3	9	1.2

**Psychiatric**				
Bipolar	72	1.3	36	4.6
Substance abuse	49	0.9	63	8.1
Schizophrenia	30	0.5	16	2.0
MDD/M	NE		41	5.2
Anorexia/bulimia	7	0.1	11	1.4
Other	7	0.1	0	0

Each condition identified in study participant was classified and listed separately; an individual may contribute more than one condition.

*Excludes subjects with missing or incomplete data

†Including strokes (45)

^‡^Including ulcerative colitis and Crohn's (34)

^§ ^Including hepatitis C (37)

NE = not evaluated; BMI = body mass index; MDD/M = major depressive disorder with melancholic features

As shown in Table 1, the CFS-like and unwell subjects seen at clinic were more likely to have exclusionary diagnoses than the well subjects, as were black individuals, those from rural and urban settings and those with a higher BMI (unadjusted ORs). However, after controlling for all other factors in Table 1, only being black and residing in a rural or urban region remained significant (adjusted ORs). As shown in Table 1 (Part B), having an exclusionary condition was associated with worse fatigue, worse impairment and more frequent and severe symptoms as measured by all subscales of the MFI, SF-36 and SI, respectively (*P *< 0.0001; unadjusted OR). However, when adjusted for all other variables in the table, only a lower (worse) vitality score (the score representing fatigue and energy) of the SF-36 was associated with exclusionary diagnoses.

The types of specific exclusionary medical and psychiatric conditions detected in clinic were similar for those otherwise classified as CFS, ISF and NF (Table 4); thyroid disease, diabetes and anaemia were in the top four medical exclusions for each group. Compared with the telephone interview, a higher proportion of exclusionary diagnoses identified in the clinic were psychiatric. However, the distribution of psychiatric diagnoses differed in that substance abuse and psychosis accounted for all of the exclusions in the NF group (57% and 7%, respectively) and the ISF group but for only 34% collectively in the CSF group where major depressive disorder with melancholic features (MDD/M) was most common (48%). Twenty-one percent of the 100 subjects who were classified as 'CFS, but for an exclusionary diagnosis', reported having been previously diagnosed as CFS by a physician.

**Table 4:** Exclusionary medical and psychiatric conditions by clinical classification

**CFS (n = 213)**		**ISF (n = 421)**		**NF (n = 147)**	
Medical conditions					

Thyroid	23.81%	Thyroid	26.76%	Anaemia	25.00%

Diabetes	19.05%	Anaemia	18.31%	Thyroid	25.00%

Anaemia	9.52%	Diabetes	16.90%	Autoimmune	15.00%

Heart	9.52%	Autoimmune	14.08%	Diabetes	10.00%

Inflammatory	9.52%	Inflammatory	4.23%	Heart	5.00%

Arthritis	7.14%	Heart	2.82%	Hematologic	5.00%

Autoimmune	7.14%	Kidney disease	2.82%	Inflammatory	5.00%

Pulmonary	7.14%	Metabolic	2.82%	Kidney disease	5.00%

Kidney disease	2.38%	Stroke	2.82%	Metabolic	5.00%

Metabolic	2.38%	Arthritis	1.41%	Arthritis	0.00%

Sleep	2.38%	Brain tumour	1.41%	Brain tumour	0.00%

Brain tumour	0.00%	Dehydration	1.41%	Dehydration	0.00%

Dehydration	0.00%	Pain syndrome	1.41%	Liver disease	0.00%

Hematologic	0.00%	Pulmonary	0.00%	Pain syndrome	0.00%

Pain syndrome	0.00%	Haematologic	0.00%	Pulmonary	0.00%

Stroke	0.00%	Liver disease	0.00%	Sleep	0.00%

-----------	------------------	Sleep	0.00%	Stroke	0.00%

Psychiatric conditions					

MDD/M	47.73%	Substance abuse	57.35%	Psychosis	50.00%

Bipolar	27.27%	Bipolar	26.47%	Substance abuse	50.00%

Substance abuse	22.73%	MDDM	14.71%	MDDM	0.00%

Psychosis	11.36%	Anorexia	10.29%	Bipolar	0.00%

Anorexia	2.27%	Psychosis	7.35%	Anorexia	0.00%

CFS = chronic fatigue syndrome; ISF = insufficient fatigue; NF = non-fatigue; MDDM = major depressive disorder with melancholic features.

### Characteristics of clinic population with medical versus psychiatric exclusions

Subjects with psychiatric exclusionary conditions were significantly younger than subjects with medical exclusions (mean age 39.9 years versus 45.4 years; *P *< 0.01; Table 5). Subjects with psychiatric conditions also had significantly higher mean scores (more fatigue) for the general fatigue and mental fatigue subscales of the MFI and non-CFS symptom inventory summary score. They also had significantly lower scores (worse functioning or more impaired) on several subscales of the SF-36 - particularly mental health, role emotional and vitality, as well as the health utility index - compared to subjects with medical conditions (*P *< 0.01 for all; Table 5). However, the fatigue lasted longer for those with medical exclusions than those with psychiatric exclusions (mean 105 months versus 60 months, *P *< 0.01; Table 5).

**Table 5:** Characteristics of subjects with exclusionary conditions by types of exclusions (n = 280)

	**Type of exclusion**						
		
	**Med (*n *= 133)**		**Psych (*n *= 114)**		**Both (*n *= 33)**		***p*-value**
	**n**	**%**	**n**	**%**	**n**	**%**	
		
**Sex**							0.0143
Female	109	81.95	75	65.79	25	75.76	
Male	24	18.05	39	34.21	8	24.25	
**Race**							0.6690
Black	48	46.09	33	28.95	10	30.30	
White	78	58.65	77	67.54	21	63.64	
Other	7	5.26	4	3.51	2	6.06	
**Residential areas**							0.3099
Rural	60	45.11	65	57.02	19	57.58	
Urban	57	42.86	35	30.70	11	33.33	
Metropolitan	16	12.03	14	12.28	3	9.09	
	Mean	SD	Mean	SD	Mean	SD	
		
Duration of fatigue (months) *^†^	105.45	96.73	59.92	59.58	120.17	92.96	0.0003
**BMI**	29.27	5.68	27.94	5.38	29.55	5.10	0.115
**MFI**							
General fatigue †‡	13.10	4.55	14.69	4.18	16.15	2.99	<0.001
Physical fatigue *‡	11.68	4.49	11.74	4.09	14.58	3.68	0.002
Reduced activity ‡	9.54	4.19	10.11	4.41	12.55	3.88	0.002
Reduced motivation ‡	9.95	3.86	10.71	4.33	12.88	3.57	0.001
Mental fatigue^†^‡	10.83	4.39	13.05	4.32	13.85	4.24	<0.001
**SF-36**							
Physical functioning*	67.86	27.04	71.36	24.82	53.33	25.15	0.002
Role physical ‡	31.20	40.17	27.41	36.28	9.85	23.33	0.013
Bodily pain	54.08	26.85	53.23	26.29	42.18	28.05	0.067
Social functioning ‡	68.80	26.72	58.44	30.87	49.24	26.51	<0.001
Mental health^†^‡	71.43	18.78	56.18	24.07	46.55	19.84	<0.001
Role emotional^†^‡	48.87	43.53	31.58	39.13	14.13	23.61	<0.001
Vitality*^†^‡	48.83	26.81	38.03	26.50	23.03	17.59	<0.001
General health*‡	57.67	24.09	56.48	22.33	41.18	24.19	0.001
PCS* ^§^	42.22	11.64	45.43	11.32	38.31	11.16	0.0040
MCS†‡^§^	46.55	11.24	37.36	14.72	33.47	10.02	<0.0001
HUI2*‡^§^	74.50	14.32	69.34	14.72	60.50	11.25	<0.0001
**Symptom scores**							
CFS symptom summary score ‡	23.98	20.70	30.13	21.14	37.98	21.90	0.0014
Non-CFS symptom summary score^†^‡	18.71	17.93	27.86	30.38	35.67	21.40	<0.0001

* Indicates the p-value for an post-hoc comparison between both and medical types with Tukey *p*-adjustment less than 0.01 for multiple group comparison.

† Indicates the *P*-value for a *post hoc *comparison between medcial and psychiatric types with Tukey *P*-adjustment less than 0.01 for multiple group comparison.

‡ Indicates the *P*-value for a *post hoc *comparison between both and psychiatric types with Tukey *P*-adjustment less than 0.01 for multiple group comparison.

§ Nichol's health utility index 2 (HUI2) [14] was included in the multivariate model because it is a linear combination of 8 SF-36 subscales and strongly correlated with Physical Component Summary (PCS) and Mental Component Summary (MCS).

Subjects with both medical and psychiatric diagnoses had higher MFI-20 scores (more fatigue) in all dimensions and these differences were statistically significant for physical fatigue, reduced activity and reduced motivation (*P *< 0.01 for all). In addition, subjects with both types of conditions had significantly lower mean scores for SF-36 subscales measuring physical functioning and role physical. Subjects with only psychiatric exclusionary conditions were significantly less impaired than those with only medical exclusionary conditions for physical functioning and in the physical component summary score (*P *< 0.01 for both). Overall, the group with both types of exclusionary diagnoses demonstrated greater fatigue, greater impairment and higher symptom scores.

### Risk factors for an exclusionary diagnosis in subjects otherwise CFS

Table 6 examines the factors associated with an exclusionary diagnosis for the 210 subjects evaluated in the clinic who fulfilled all other criteria for a diagnosis of CFS. Unadjusted OR for BMI, higher scores (worse) in the reduced motivation subscale of the MFI-20, lower scores (worse) in the physical functioning, role physical, HU12 subscales of the SF-36 and the non-CFS symptom scores of the symptom inventory all showed increased risks of exclusion. However, after adjusting for all other variables in the table, only the BMI remained significantly associated with having an exclusionary condition, with a 47% increased risk for exclusion in subjects with a standard deviation increment (5.13 kg/m^2^) of BMI (OR_adj _= 1.47, 95% confidence interval = 1.05, 2.06).

**Table 6:** Factors associated with the presence of exclusionary condition among subjects classified as chronic fatigue syndrome (CFS) at clinic evaluation

**Characteristics**			**Exclusionary condition**				**Odds ratio (OR)**	
	**All**		**Yes**		**No**		**Unadjusted ORs**	**Adjusted ORs**
	***n *= 210**		***n *= 100 (48%)**		***n *= 110 (52%)**		**(95% CI)**^**†**^	**(95% CI)**
Telephone Classification								
CFS-like	169	80.48	85	50.30	84	49.70	1.75 (0.87-3.54)	1.48 (0.61-3.56)
Unwell	41	19.52	15	36.59	26	63.41	1.00	1.00
Age (years), mean (standard deviation, SD)	45.06	(9.65)	45.99	(8.98)	44.22	(10.19)	1.21 (0.92-1.59)	1.06 (0.74-1.54)
Sex								
Male	38	18.10	18	47.37	20	52.63	0.99 (0.49-2.00)	1.65 (0.69-3.92)
Female	172	81.90	82	47.67	90	52.33	1.00	1.00
Race								
Black	35	16.67	14	40.00	21	60.00	0.67 (0.32-1.40)	0.56 (0.20-1.52)
Other	13	6.19	5	38.46	8	61.54	0.63 (0.20-1.99)	0.62 (0.15-2.51)
White	162	77.14	81	50.00	81	50.00	1.00	1.00
Residential areas								
Rural	106	50.48	54	50.94	52	49.06	2.28 (0.99-5.29)	1.47 (0.51-4.20)
Urban	72	34.29	36	50.00	36	50.00	2.20 (0.91-5.30)	1.53 (0.55-4.27)
Metropolitan	32	15.24	10	31.25	22	68.75	1.00	1.00
Body mass index (kg/m^2^), mean (SD)	28.69	(5.18)	29.50	(5.42)	27.95	(4.87)	**1.36 (1.03-1.79)**	**1.47 (1.05-2.06)**
Multidimensional fatigue inventory							Per SD increment	Per SD increment
General fatigue	17.05	(2.36)	17.34	(2.09)	16.79	(2.57)	1.27 (0.96-1.70)	1.22 (0.81-1.84)
Physical fatigue	14.52	(3.31)	14.88	(3.22)	14.19	(3.38)	1.24 (0.94-1.63)	0.83 (0.52-1.34)
Reduced Activity	11.96	(4.10)	12.33	(4.19)	11.63	(4.02)	1.19 (0.90-1.56)	0.83 (0.54-1.26)
Reduced Motivation	12.44	(3.38)	13.05	(3.63)	11.89	(3.05)	**1.43 (1.07-1.89)**	1.44 (0.94-2.20)
Mental fatigue	14.05	(3.75)	14.49	(3.72)	13.65	(3.75)	1.25 (0.95-1.65)	1.19 (0.80-1.77)
SF-36							Per SD increment	Per SD increment
Physical functioning	57.37	(24.54)	52.83	(24.01)	61.45	(24.40)	**0.70 (0.53-0.92)**	0.77 (0.28-2.12)
Role physical	9.05	(19.45)	5.50	(13.09)	12.27	(23.40)	**0.67 (0.48-0.93)**	0.63 (0.40-1.00)
Bodily pain	38.30	(19.86)	35.66	(18.93)	40.70	(20.46)	0.77 (0.58-1.02)	0.60 (0.27-1.31)
Social functioning	49.82	(24.74)	47.25	(26.03)	52.16	(23.39)	0.82 (0.62-1.08)	1.24 (0.62-2.46)
Mental health	54.27	(22.15)	52.04	(21.59)	56.29	(22.55)	0.82 (0.63-1.08)	1.38 (0.55-3.46)
Role emotional	27.46	(37.36)	23.66	(35.23)	30.90	(39.03)	0.82 (0.62-1.08)	0.94 (0.55-1.61)
Vitality	22.29	(16.21)	21.55	(16.96)	22.95	(15.54)	0.92 (0.70-1.20)	1.20 (0.72-1.99)
General health	40.17	(19.46)	38.10	(20.87)	42.05	(17.97)	0.81 (0.62-1.07)	0.99 (0.51-1.92)
PCS*	36.67	(9.92)	35.31	(9.91)	37.91	(9.81)	0.77 (0.58-1.01)	1.47 (0.17-12.75)
MCS*	36.75	(12.58)	35.75	(12.78)	37.67	(12.39)	0.86 (0.65-1.13)	0.69 (0.12-3.99)
HUI2*	61.94	(10.80)	59.95	(10.96)	63.76	(10.37)	**0.69 (0.52-0.92)**	Not included
SI							Per SD increment	Per SD increment
SI_CFS	49.03	(16.60)	50.20	(15.94)	47.97	(17.17)	1.15 (0.87-1.50)	0.68 (0.42-1.11)
SI_NCFS	36.98	(18.68)	39.95	(19.63)	34.24	(17.40)	**1.37 (1.03-1.82)**	1.47 (0.93-2.32)

Note: adjusted ORs were obtained for the multiple logistic regression models including all variables in the table; p-value < 0.05 were in bold font.

* Nichole's health utility index 2 (HUI2) was included in the multivariate model because it is a linear combination of 8 SF-36 subscales and strongly correlated with Physical Component Summary (PCS) and Mental Component Summary (MCS).

CI: confidence interval; SI_CFS: symptom inventory summary score of 8 CFS symptoms; SI_NCFS: symptom inventory summary score of non-CFS symptoms

## Discussion

The study illustrates the complexity that exclusionary conditions introduce into both research and clinical considerations of subjects potentially classified as CFS. The exclusionary diagnoses are common; 28% of subjects in a population-based sample had exclusions identified during a personal history telephone interview. Exclusionary conditions/diseases were subsequently recognized in an additional 36% of subjects undergoing a full clinical examination. It appears that during the telephone interview subjects did not know of or did not remember all of their exclusionary diagnoses. This discrepancy could be due to: (1) greater accuracy and specificity of information obtained by clinical examination compared to telephone interview; (2) variable access to healthcare which, in turn, means variable access to diagnostic means [16]; (3) asymptomatic status contributing to unrecognized conditions; or (4) a lack of perception of bodily feelings as being abnormal. The types of diseases identified in the clinic are recognized to be important in the differential diagnosis of CFS and emphasize the need for clinicians to evaluate patients with appropriate history taking, physical examination and laboratory tests. The finding that Black people and residents of rural and urban areas were more likely to have exclusionary conditions diagnosed at a clinic suggests that access to healthcare may be a contributing factor, as limited access to quality healthcare has been documented among these subgroups [16]

The finding that increased BMI was independently associated with having an exclusionary condition among CFS patients, suggests that the condition may be 'silent' as in some correlates of elevated BMI, including metabolic conditions, sleep disorders and hypothyroidism. This interpretation is supported by the finding of exclusionary conditions in 26 people who, in the telephone interview, had considered themselves 'well'.

The diagnoses considered exclusionary for CFS were originally selected because they frequently result in symptoms similar, if not identical, to those characteristic of CFS subjects. As might be expected, differences between subjects with and without exclusionary diagnoses were not universally present. For example, general fatigue and reduced activity scores of the MFI and physical function, role physical, social function and role emotional scores of the SF-36 were equivalent. Likewise, CFS and non-CFS symptom scores were not significantly different. Thus, in terms of level of overall 'sickness' [17] and impairment, included and excluded subjects were comparable. It is not surprising that subjects with both medical and psychiatric diagnoses have more fatigue and impairment, as well as higher symptom scores, than those with only medical or psychiatric conditions. However, greater impairment in selected parameters in those subjects with only psychiatric exclusions versus those with only medical exclusions reflects a true difference or bias introduced by the self-report procedure.

Although not emphasizing the differences between subjects who fulfill defined requirements for exclusionary diagnoses and those who do not, previous studies of chronic fatigue and CFS have shown high rates of psychiatric morbidity and functional morbidity and have documented these outcomes as important public health burdens [18,19]. Another study, which included 98 subjects with chronic fatigue and compared disability and psychosocial distress in those that met criteria for CFS and those who had medical or psychiatric exclusions, failed to meet the definition or were using medications specific to the study [20]. The study results showed that the CFS subjects could not be differentiated from those who were excluded based on the study variables that addressed symptoms of depression, general health, impairment, symptom perception and somatic and psychological stress.

In principle, the results of this study, and of previous observations, support the original decision to exclude subjects with fatigue and selected concurrent identifiable illnesses/diseases from the research diagnosis of CFS as the latter shares illness characteristics and consequences with the syndrome [1]. The excluded group may have exposures or disease components that would confound efforts to address the incidence, prevalence or pathophysiology of an otherwise unexplained condition such as CFS if it were unique [21,22]. We did not address the question of subject competency to complete the in the psychiatric exclusions. Thus, considering CFS as a diagnostic possibility and pursuing a differential diagnostic process should allow identification of patients who need careful evaluations as is recommended in the 1994 definition. In particular, obesity, anaemia, thyroid disease, diabetes and heart disease are common in fatigued as well as non-fatigued subjects (Table 3 and 4).

It is equally clear that subjects with exclusionary diagnoses are at least as comparably functionally impaired as the included subjects. The chronically unwell population, identified here with a fatiguing illness, is likely to have a medical disease and/or a psychiatric disease. However, these conditions may go undiagnosed. Since subjects with and without identifiable exclusionary disease processes share many symptoms, clinical management requires careful attention in order to correctly identify and treat the medical and psychiatric illnesses. It is clear that treatment of the underlying diseases will not resolve fatigue and symptoms in all instances and it is possible that illnesses/diseases with chronic fatigue share a common underlying pathophysiologic mechanism. In order to examine this possibility, subjects with exclusionary conditions could be included with CSF cases for comparison.

The primary limitation of this study is the inclusion of subjects who have been ill for an average of 6-7 years. The majority of the subjects had experienced a gradual onset of their illness. Thus the exclusion of subjects fulfilling CFS criteria and having medical and/or psychiatric illnesses may be observed more frequently in the population under study than in the younger individuals with an acute onset. Likewise, the levels of impairment may also be more applicable to this population. Replication of these observations will be possible in future follow-up studies.

As those with CFS suffer from personal, social, workplace [1] and observed financial losses [23], should not all individuals fulfilling CFS inclusion criteria, with or without exclusionary diagnoses, be considered in future public health planning? For instance, would both groups benefit from prevention and intervention efforts such as cognitive behavioral therapy and graded exercise therapy [24,25]? A similar question could be asked of those who are unwell but who do not reach the diagnostic threshold.

## Conclusion

This analysis allows three different, but complementary, interpretations of the results that need to be addressed in future studies. First, it substantiates the need for the identification of underlying diagnoses that impact the choice of subjects for epidemiological and mechanistic studies of a specifically described illness construct. Secondly, it suggests that those who would otherwise fulfill the CFS definition, but who have an exclusionary illness or disease, are as impaired as those defined as having CFS. The third interpretation is that CFS, in the population studied here, may reflect underlying medical or psychiatric illnesses or diseases that are not necessarily 'under control'. The data therefore suggest that, in order to substantiate CFS as a specific entity or to identify shared pathophysiological factors, subjects fulfilling the criteria of fatiguing illnesses, but who have exclusionary and non-exclusionary diagnoses, need to be included as comparison case groups in both mechanistic and therapeutic studies. The identification of subjects for CFS studies requires a clinical evaluation; medical and psychiatric histories obtained by telephone interviews are not completely reliable.

## Abbreviations

BMI: body mass index; CATI: computer-assisted telephone interview; CDC: US Centers for Disease Control and Prevention; CFS: chronic fatigue syndrome; DSM: *Diagnostic and statistical manual of mental disorders*; DSM-IV: disorders described in the fourth edition of the DSM; ISF: insufficient fatigue; MDDM: major depressive disorder with melancholic features; MFI: Multidimensional Fatigue Inventory; MCS: mental component summary; NF: non-fatigued; OR: odds ratio; PCS: physical component summary; SCID: structured clinical interviews for DSM disorders; SF-36: short form 36; SI: symptom inventory.

## Competing interests

The authors declare that they have no competing interests.

## Authors' contributions

JFJ, ERU and WCR were involved in the conception and design. JMSL, EMM, RSB, UMN, ERU, JFJ and WCR were involved in the analysis and interpretation of the data. JFJ. JMSL, EMM, ERU, RSB and WCR drafted the article. JFJ, JMSL, EMM, RSB, UMN, ERU and WCR were responsible for critical revision of the article for intellectual content. JFJ, JMSL, EMM, RSB, UMN, ERU and WCR gave final approval of the article. JMSL, EMM and WCR provided statistical expertise. JMSL, JFJ and WCR were involved in the collection and assembly of the data.

## Pre-publication history

The pre-publication history for this paper can be accessed here:


